# Computational Methods for the Pharmacogenetic Interpretation of Next Generation Sequencing Data

**DOI:** 10.3389/fphar.2018.01437

**Published:** 2018-12-04

**Authors:** Yitian Zhou, Kohei Fujikura, Souren Mkrtchian, Volker M. Lauschke

**Affiliations:** ^1^Section of Pharmacogenetics, Department of Physiology and Pharmacology, Karolinska Institutet, Stockholm, Sweden; ^2^Department of Diagnostic Pathology, Kobe University Graduate School of Medicine, Kobe, Japan

**Keywords:** precision medicine, personalized medicine, variant effect prediction, ADME, NGS, rare variant analysis, noncoding variation, pharmacogenomics

## Abstract

Up to half of all patients do not respond to pharmacological treatment as intended. A substantial fraction of these inter-individual differences is due to heritable factors and a growing number of associations between genetic variations and drug response phenotypes have been identified. Importantly, the rapid progress in Next Generation Sequencing technologies in recent years unveiled the true complexity of the genetic landscape in pharmacogenes with tens of thousands of rare genetic variants. As each individual was found to harbor numerous such rare variants they are anticipated to be important contributors to the genetically encoded inter-individual variability in drug effects. The fundamental challenge however is their functional interpretation due to the sheer scale of the problem that renders systematic experimental characterization of these variants currently unfeasible. Here, we review concepts and important progress in the development of computational prediction methods that allow to evaluate the effect of amino acid sequence alterations in drug metabolizing enzymes and transporters. In addition, we discuss recent advances in the interpretation of functional effects of non-coding variants, such as variations in splice sites, regulatory regions and miRNA binding sites. We anticipate that these methodologies will provide a useful toolkit to facilitate the integration of the vast extent of rare genetic variability into drug response predictions in a precision medicine framework.

## Introduction

Inter-individual differences in drug response are clinically important phenomena that result in reduced efficacy or adverse reactions in 25–50% of all patients and genetic factors have been estimated to account for around 20–30% of these (Spear et al., 2001; Sim et al., 2013). Fueled by technological advances in Next-Generation Sequencing (NGS) technologies, the application of comprehensive sequencing approaches is on the rise for various applications, including studies of biodiversity, population genetics and biomedical research (Levy and Myers, 2016). Furthermore, plummeting costs to < 1,000 USD per human genome and increasing worldwide sequencing capacities that we estimate to exceed 100 petabases per year (10^15^ bases corresponding to the size of around 100,000 human genomes) open tremendous possibilities for NGS to revolutionize precision medicine.

Strikingly, these massive NGS data sets revealed that individuals harbored on average more than 3.7 million single nucleotide variants (SNVs) and more than 350,000 insertions and deletions across different populations, emphasizing the substantial variability of the human genome (The 1000 Genomes Project Consortium, 2012). Particularly genes involved in drug absorption, distribution, metabolism and excretion (ADME) proved to be highly diverse and genetically complex (Fujikura et al., 2015; Bush et al., 2016; Kozyra et al., 2017). Across 208 ADME genes more than 69,000 SNVs have been described, 98.5% of these being rare with minor allele frequencies (MAF) < 1% (Ingelman-Sundberg et al., 2018). The overall pharmacogenetic variability was highly population specific, particularly for isolated populations, such as Ashkenazi Jews (Ahn and Park, 2017; Kozyra et al., 2017; Zhou and Lauschke, 2018). Given this enormous pharmacogenetic variability, one of the key frontiers of contemporary pharmacogenomics is the translation of these comprehensive genomic data into clinically actionable treatment recommendations (Lauschke and Ingelman-Sundberg, 2016a, 2018).

Heterologous expression in cell lines followed by quantitative determination of gene product functionality using appropriate end points is considered as the gold standard strategy to characterize the functional impact of pharmacogenetic variants. Furthermore, epidemiological association studies can provide additional indications about the consequences of genetic variants on drug metabolism related phenotypes *in vivo*. However, for the functional interpretation of rare variants these approaches suffer from multiple shortcomings:

- These methods are generally low throughput and are not compatible with the interrogation of tens of thousands of variants.
- Experimental characterizations are time consuming, expensive and require specially trained technical staff, which renders them unsuitable for the rapid functional interpretation of the pharmacogenotype of an individual patient at the point of care.
- Epidemiological analyses require a sufficient number of patients who carry the allele, which drastically limits their feasibility for rare genetic variant studies.

**Figure 1:**
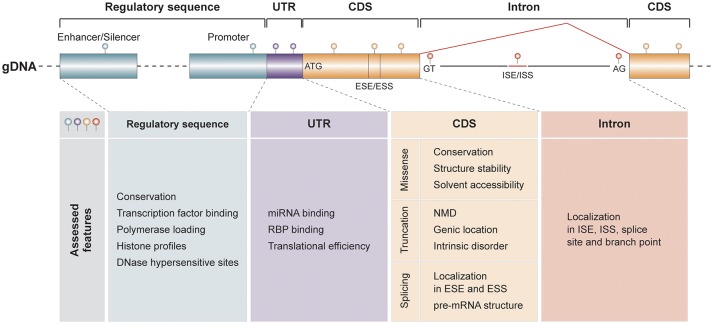
Overview of features that can be assessed by current computational prediction methods. Different parameters and features are assessed for genetic variants depending on whether they are localized in putatively regulatory sequences, untranslated regions (UTR) of the gene, its coding sequences (CDS) or within introns. ESE/ESS, exonic splicing enhancer/silencer; ISE/ISS, intronic splicing enhancer/silencer; NMD, nonsense-mediated decay; RBP, RNA binding protein.

Thus, in the absence of viable experimental strategies, computational prediction methodologies are routinely used to predict the functional impact of genetic variants. Most of these algorithms focus on predicting the functional consequences of variants that result in amino acid substitutions. However, recently much progress has also been made regarding the interpretation of non-coding variants that affect splice sites, promoters, enhancers or miRNA binding sites (Figure 1).

Prediction algorithms are generally trained on pathogenic variant sets and most tools base their conclusions, at least in part, on the evolutionary conservation of the respective sequence. Importantly however, pharmacogenes are hallmarked by low evolutionary conservation and are generally not associated with human disease. These peculiarities result is specific problems for the interpretation of pharmacogenetic variants. Here, we provide an updated overview of computational approaches for the functional interpretation of genetic variants, specifically focusing on their suitability for pharmacogenetic predictions. We describe the underlying statistical frameworks and discuss their different bases for decision-making. Furthermore, we highlight important progress particularly in the interpretation of non-coding genetic variability. We conclude that computational tools are essential for the functional interpretation of an individual's pharmacogenotype and that their further improvement constitutes one of the most important frontiers for the clinical implementation of NGS-based genotyping.

## Interpretation of Variants Resulting in Amino Acid Exchanges

Genetic variants that result in amino acid substitution, henceforth termed missense variants, can impact the functionality of the respective protein by various mechanisms, including alterations in active sites, structural destabilization due to protein misfolding, perturbations in solvent accessibility or modification of post-translational processing. Each individual harbors 10,000–12,000 missense variants, many of which are rare (The 1000 Genomes Project Consortium, 2015). These rare variants have been suggested as important modulators of complex disease risk (Kryukov et al., 2007) and inter-individual differences in drug response (Kozyra et al., 2017). Among all variant classes, missense variants are the most extensively studied and a plethora of computational methods is available for their functional interpretation. Conceptually, these algorithms predict the functional impact of missense variants based on sequence information, primarily evolutionary conservation of the respective residues, and/or structural information of the corresponding gene product. In the following, we highlight recent progress, provide an overview of available tools and discuss their utility for pharmacogenetic predictions. For methodological details we refer the interested reader to excellent recent reviews (Ng and Henikoff, 2006; Peterson et al., 2013; Tang and Thomas, 2016).

### Predictions Based on Sequence Information

Evolutionary conservation scores are calculated by analyzing the evolutionary variation dynamics of DNA or amino acid sequences among homologs with the hypothesis that the extent of conservation is a strong predictor of the importance of the respective sequence for structure and function of the corresponding gene product. Thus, positions with a high evolutionary rate are thought to be dispensable, whereas slowly evolving, i.e., conserved sequences indicate a selective pressure against variation in these regions and thus deleterious effects if mutated.

Evolutionary conservation as a metric to distinguish deleterious from neutral variants is considered by most computational prediction algorithms. The majority of approaches that focus on the functional interpretation of missense variants utilize amino acid sequence alignment, whereas others utilize nucleotide sequence alignments or a combination of both methods (Table 1). While alignment of amino acid sequence proved to be effective for the analysis of missense variants, genomic sequence alignments provide additional versatility and allow to extend functional interpretations to variant classes that do not alter the amino acid sequence, such as synonymous and regulatory variants. Notably, commonly used conservation-based functionality predictors do not consider sequence interdependencies. Explicit integration of residue dependency information obtained from multiple sequence alignments was however recently shown to improve predictive performance (Hopf et al., 2017), emphasizing the added value of complementing conservation based functionality predictions with variant interaction data.

**Table 1:** Methods to predict the functional effect of missense variants based on sequence information.

**Algorithm**	**Model**	**Basis of decision**	**Model training or evaluation**	**References**
SIFT	Direct	Prediction of functionality based on sequence conservation metrics that make use of Dirichlet priors	Variants from protein specific studies (LacI, HIV-1 Protease and Bacteriophage T4 Lysozyme)	Ng and Henikoff, 2001
PANTHER	HMM	Sequence conservation analysis using HMM	Variants from HGMD and dbSNP as deleterious and functionally neutral variants, respectively	Thomas et al., 2003
MAPP	Direct	Quantification of the physicochemical characteristics at each position of the amino acid sequence based on observed evolutionary variation	Protein specific studies (LacI, HIV-1 Protease, HIV reverse transcriptase and Bacteriophage T4 Lysozyme)	Stone and Sidow, 2005
PhastCons	HMM	Identification of conserved elements using a two-state phylogenetic HMM	Calibration on genomes from four model species (human, D. melanogaster, C. elegans, and S. cerevisiae)	Siepel et al., 2005
SNPs3D	SVM	Variant effect prediction based on amino acid sequence conservation metrics and folded state stability of protein structure	Variants from HGMD and dbSNP as deleterious and functionally neutral variants, respectively	Yue et al., 2006
PhD-SNP	SVM	Prediction of variant pathogenicity based on sequence profiles	Variants from HumVar and HumVarProf datasets	Capriotti et al., 2006
SiPhy	HMM	Sequence conservation analysis using HMM	ENCODE Phase I regions	Garber et al., 2009
LRT	Direct	Evolutionary conservation model across 32 vertebrates	Variants in three sequenced human genomes	Chun and Fay, 2009
SNPs&GO	SVM	Variant effect prediction based on sequence information, evolutionary conservation and defined gene ontology score	Variants from SwissProt	Calabrese et al., 2009
B-SIFT	Direct	Sequence conservation metrics that calculate the difference between wild-type and mutant allele	Variants from SwissProt database and protein specific study (Dnase I)	Lee et al., 2009
PolyPhen-2	NB	Considering sequence conservation, Structure parameters such as hydrophobic propensity and B factor	Variants fromn HumDiv and HumVar from UniProt Database	Adzhubei et al., 2010
MutationTaster	NB	Prediction of mutation pathogenicity based on evolutionary conservation, splice-site changes, loss of protein features and changes that affect expression levels	Variants from OMIM database, HGMD and the literature as pathogenic set and neutral variants from dbSNP as controls	Schwarz et al., 2014
MutationAssessor	Direct	Evolutionary conservation patterns within protein families and across species using combinatorial entropy	Variants from UniProt database (HumSaVar)	Reva et al., 2011
Condel	Direct	Integration of five algorithms (SIFT, PolyPhen-2 MAPP, MutationAssessor, and Log R Pfam E-value) into single output score	Variants from HumVar, HumDiv, Cosmic database, IARC TP53 database	González-Pérez and López-Bigas, 2011
PROVEAN	Direct	Alignment-based score that can also assess in-frame insertions, deletions, and multiple amino acid substitutions	Missense variants and indels, replacements from UniProt database	Choi et al., 2012
FATHMM	HMM	Identification of pathogenic variants based on sequence conservation, protein domain-based information and species-specific pathogenicity weights. Also suitable for prediction of non-coding variations.	Variants from the HGMD and Uniprot databases	Shihab et al., 2013, 2015; Rogers et al., 2018
VEST	RF	Prioritization of variants underlying Mendelian diseases	Rare variants from HGMD database as pathogenic set and variants from ESP	Carter et al., 2013
Evolutionary Action	Direct	Prediction of variant effects on evolutionary fitness using a formal genotype-phenotype perturbation equation	Variants from 1000 Genomes Project	Katsonis and Lichtarge, 2014
MetaSVM	SVM	Ensemble score integrating nine functionality predictors (SIFT, PolyPhen-2, GERP++, MutationTaster, MutationAssessor, FATHMM, LRT, SiPhy and PhyloP)	Variants causing Mendelian diseases as pathogenic set and variants that are not associated with any phenotypes as controls, all from Uniprot database	Dong et al., 2015
MetaLR	RM	Same as MetaSVM but using logistic regression instead of SVM.		Dong et al., 2015
SuSPect	SVM	Sequence conservation metrics, structure features and additional network information	Variants from Humsavar database	Yates et al., 2014
PredictSNP	EL	Ensemble score integrating six functionality predictors (MAPP, PhD-SNP, PolyPhen-1, PolyPhen-2, SIFT and SNAP)	Variants mainly from SwissProt, HGMD, dbSNP and Humsavar database	Bendl et al., 2014
SNAP2	NN	Prediction of amino acid variations based on amino acid properties, predicted binding residues, predicted disordered and low-complexity regions, proximity to N- and C-terminus, statistical contact potentials, co-evolving positions, secondary structure and solvent accessibility	Variants from PMD, Swiss-Prot, OMIM, HumVar and protein specific data sets (LacI)	Hecht et al., 2015
REVEL	RF	Ensemble method tailored specifically for the prediction of rare genetic variant effects integrating MutPred, FATHMM, VEST, PolyPhen, SIFT, PROVEAN, MutationAssessor, MutationTaster, LRT, GERP, SiPhy, phyloP, and phastCons	Variants from HGMD as pathogenic set and neutral variants from ESP as controls	Ioannidis et al., 2016
ConSurf	Empirical Bayesian method and maximum likelihood estimation	Mapping of evolutionarily conserved residues on protein surfaces by estimating the evolutionary rates of each nucleic acid and amino acid sequence position using multiple sequence alignments. Also offers RNA secondary structure predictions.	Protein with at least five known 3D structure homologs and precise annotation of their functional sites (with different nature)	Ashkenazy et al., 2016
VIPUR	RM	Combination of sequence- and structure-based features to identify and functionally interpret deleterious variants	Variants from HumDiv and UniProt with clear evidence of protein disruption	Baugh et al., 2016
Envision	GTB	Decision tree ensemble-based tool using a stochastic gradient boosting learning algorithm	Variants from nine large-scale experimental mutagenesis datasets in eight proteins	Gray et al., 2018
EVmutation	Direct	Unsupervised method exploiting sequence conservation by incorporating interaction information between all pairs of residues in protein	34 data sets from 21 proteins and a tRNA gene extracted from 27 publications	Hopf et al., 2017
PredSAV	GTB	Identification of pathogenic variants based on sequence, structure, residue-contact networks as well as structural neighborhood features	Human variants from Uniprot and OMIM as pathogenic set and Ensemble variants as neutral controls	Pan et al., 2017
SNPMuSiC	NN	Structure stability based, implement PoPMuSiC and HoTMuSiC on the basis of 13 statistical potentials (distence potentials, solvent accessibility potentials and torsion potentials) and 2 biophysical characteristics (solvent accessibility of mutated residue and difference in volume)	Variants from dbSNP, SwissVar and HumSaVar datasets	Ancien et al., 2018
DEOGEN2	RF	Integration of 11 scores and metrices into one meta-score, considering evolutionary features, folding predictions, domain information as well as gene features to identify deleterious variants	Training and test on variants from the UniProt Humsavar16 dataset	Raimondi et al., 2017
ADME prediction framework	Direct	Integration of prediction scores from five orthogonal algorithms (LRT, MutationAssessor, PROVEAN, VEST3 and CADD) using parameters optimized for pharmacogenes	Training and validation specifically on experimentally characterized pharmacogenetic data sets from 43 ADME genes	Zhou et al., 2018

*HMM, hidden Markov model; SVM, support vector machine; NB, naïve Bayes classifier; EL, ensemble learning; RF, random forest; RM, regression model; NN, neural networks; GTB, gradient tree boosting; HGMD, Human Gene Mutation Database; OMIM, Online Mendelian Inheritance in Man; ESP, Exome Sequencing Project; PMD, Protein Mutant Database*.

On the basis of multiple sequence alignments, algorithms derive their functionality predictions either based on direct theoretical models, or by various machine-learning approaches. The former methods predict the functional impact of variants based on phenomenological scores derived from theoretical models that are known *a priori*. In contrast, machine learning methods search for patterns in multi-dimensional training data sets consisting of labeled deleterious and benign variations, which will then be used as the basis to generate predictions on new unlabeled data. Machine learning approaches include support vector machines, random forests, artificial neural networks, naive Bayes approaches, gradient tree boosting and regression models. With increasing wealth of large-scale data sets to learn from, machine learning methods become increasingly popular as versatile tools to generate predictive models in many areas of biomedicine (Camacho et al., 2018).

Commonly used algorithms are generally designed to flag deleterious variants, which are mostly assumed to result in a reduced gene product function, and their performance of gain-of-function variants is substantially worse (Flanagan et al., 2010). Notably, the algorithm B-SIFT, a modified version of the widely used SIFT tool (Ng and Henikoff, 2001), was developed to overcome this limitation (Lee et al., 2009). Conceptually, B-SIFT identifies increased functionality variants based on protein sequence alignments by scoring whether a given mutation results in a change commonly present in protein homologs and the tool successfully identified experimentally validated gain-of-function variants in cancer.

While computational missense variant predictors are generally reported to achieve high predictive accuracies with areas under the receiver operating characteristic curve (AUC_ROC_) that often pivot around 0.9, drastic drops in performance to AUC_ROC_ of 0.5–0.75 have been reported on independent, functionally determined human variant datasets (Mahmood et al., 2017). These findings were corroborated by a recent cross-comparison of 23 methods based on three independent pathogenicity datasets in which the authors found that REVEL and VEST3 performed overall best, whereas the most commonly used methods SIFT and PolyPhen-2 performed only medially (Li et al., 2018). Furthermore, no functional consequences could be detected using various *in vitro* or *in vivo* tools for 40% of variants predicted to be deleterious by common functionality prediction tools (Miosge et al., 2015). Thus, while current tools have proven powerful in clinical diagnostics to prioritize potentially causative mutations in genetic diseases for further analyses (Boycott et al., 2013), their predictive power is not yet sufficient to predict functional variant effects without substantial subsequent validations.

Importantly, the quality of prediction models critically relies on accurate training data sets. For instance, models are commonly generated using training sets of pathogenic variants as positive controls and polymorphisms identified to be common in large-scale sequencing projects as negative, i.e., functionally neutral variants. For pharmacogenetic predictions such a strategy is associated with multiple problems: Firstly, training on disease-associated data sets will, in the best case, result in prediction models that accurately predict the pathogenicity of variants. However, only very few ADME genes are directly associated with disease, suggesting that pathogenicity is not the right endpoint to inform about variant effects in the pharmacogenetic arena. Secondly, while evolutionary conservation constitutes a useful metric to predict functional consequences in genes under purifying selection, evolutionary conservation in pharmacogenes is generally much lower (Fujikura, 2016), indicating that conservation cannot reliably inform about functional impacts of variations in pharmacogenes. Finally, the choice of common polymorphisms as neutral training sets is problematic. Genetic variants that occur with high frequencies are not necessarily functionally neutral, particularly in pharmacogenetic loci, as evidenced by a multitude of high-frequency loss of function variants in *CYP* genes, such as CYP3A5^*^3 (MAF = 95% in Europeans), CYP2C19^*^2 (MAF = 34% in South Asians) and CYP2D6^*^4 (MAF = 16% in Latinos) (Zhou et al., 2017).

The indicated problems incentivized us to develop a prediction framework tailored specifically toward pharmacogenetic functionality assessment (Zhou et al., 2018). Specifically, the model was devised using a two-step procedure: Firstly, functionality classification threshold of 18 commonly used functional prediction algorithms were optimized by leveraging a dataset of 337 experimentally characterized pharmacogenetic variants using 5-fold cross validations. In a second step, we integrated the best performing orthogonal algorithms following a strategy that had been shown to further improve predictive accuracy (Martelotto et al., 2014). The resulting method achieved 93% for both sensitivity and specificity for both loss-of-function and functionally neutral variants. Moreover, the returned score can provide quantitative estimates of the effect of the variant in question on gene function, thus facilitating the functional and personalized interpretation of an individual's NGS-based pharmacogenome.

Recent progress in large-scale experimental mutagenesis screens provides a promising approach to further expand the development of powerful training resources for missense variant effect predictors. While such a strategy has already been used to develop a prediction method based on 10 proteins from different species with disparate structures (Gray et al., 2018), we propose that deep mutational scanning data from ADME proteins is likely to substantially refine the resulting model for pharmacogenetic predictions. For such an endeavor, we recommend to use multiple substrates for each protein, as correlations between prediction and experiments improved with more comprehensive interrogation of protein function (Gallion et al., 2017). Combined with ADME-optimized prediction models, we envision that such an approach can further enhance the predictive accuracy of *in silico* methods and yield sufficiently accurate tools to allow for the clinical implementation of computational pharmacogenetic predictions.

### Utilization of Structural Data

While evolutionary conservation scores can provide useful metrics to assess the pathogenicity of missense variants, they have limitations when applied to the less conserved genes, such as most ADME genes, which prompted the search for additional orthogonal *in silico* methods. To this end, the analysis of predicted or experimental structural data provides an appealing concept, as the correct folding of polypeptide chains into three-dimensional tertiary structures is of paramount importance for their biological functions. Structure-based approaches either directly use known crystal or NMR structures, preferably at high resolution < 2–3 Å (Wlodawer et al., 2008) or, should such data not be available, leverage knowledge of the experimental 3D structures of homologous sequences (Table 2).

**Table 2:** Methods to predict the functional effect of missense variants based primarily on structural features.

**Algorithm**	**Model**	**Basis of decision**	**Model training or evaluation**	**References**
SDM	Direct	Predicts variant effects on thermal protein stability using conformationally constrained environment-specific substitution tables derived from 2,054 protein family sequence and structure alignments from the TOCCATA database	Validated on 2,690 SNVs from 132 different protein structures.	Topham et al., 1997; Pandurangan et al., 2017
I-Mutant	SVM	Protein structure or sequence-based prediction of point mutation effects on protein stability	Training and testing on thermodynamic experimental data of free energy changes of protein stability upon mutation from the ProTherm database	Capriotti et al., 2005
HOPE	Direct	Analyzes the structural and functional effects of point mutations based on available crystal structures, homology modeling and sequence information.	Evaluated using case studies.	Venselaar et al., 2010
mCSM	RM	Translation of distance patterns between atoms into graph-based signatures providing data that is complementary to potential energy based approaches	Prediction of protein stability changes, protein-protein and protein-nucleic acid interactions and pathogenicity based on an array of preexisting experimental data sets	Pires et al., 2014b
DUET	SVM	SVM predictor that integrates mCSM and SDM in a consensus prediction	Benchmarking again mCSM and SDM alone on p53 data set.	Pires et al., 2014a
STRUM	GTB	Predicts variant effects on protein stability based on 3D models constructed by iterative threading assembly refinement simulations	Evaluated on 3,421 experimentally determined mutations distributed across 150 proteins.	Quan et al., 2016
ELASPIC	GTB	Predicts effects of mutations on protein folding and protein–protein interactions using homology modeling of domains and domain–domain interactions	Performance analysis via case study using EP300 mutations found in COSMIC	Witvliet et al., 2016
SAAFEC	RM	Prediction of effects of amino acid changes on folding free energy using a Molecular Mechanics Poisson-Boltzmann approach	Training and testing on thermodynamic experimental data of free energy changes of protein stability upon mutation from the ProTherm database	Getov et al., 2016

*SVM, support vector machine; RM, regression model; GTB, gradient tree boosting*.

The effect of variants is predicted by how the folding free energy difference between the unfolded and folded states (ΔG°) is modified upon point mutations (ΔΔG°) with negative and positive values of ΔΔG° indicating destabilizing and stabilizing mutations, respectively. In recent years a large number of mechanistically diverse approaches have been presented, with machine learning-based strategies being most prevalent. SDM constitutes a statistical potential energy function that can estimate variant effects on protein stability (Topham et al., 1997). This approach pioneered the knowledge-based prediction of mutation effects on protein stability and has also been successfully used in combination with machine learning techniques (Pires et al., 2014a). An updated version of the tool, SDM2 (Pandurangan et al., 2017), with a 5-fold increase in underlying structural information as well as extensions for interaction modeling can be accessed through a free, publically available web server interface. Similarly, the algorithm HOPE (Venselaar et al., 2010) can calculate structural and functional effects of amino acid exchanges based on homology modeling. It should be however noted that most of the current tools are strongly biased toward the detection of destabilizing effects (Pucci et al., 2018).

Approximately 70% of the human proteome can be structurally modeled by homology (Somody et al., 2017). Yet, the number of resolved 3D structures for genes involved in drug ADME remains relatively low, at least in part due to the membrane bound nature of many of these proteins. Furthermore, as many metabolic enzymes, such as cytochrome p450s (CYPs) exhibit marked active-site flexibility, which often results in ligand-induced conformational changes, prediction of variant effects based on direct structural data is difficult for these proteins and substrate-specific effects have to be considered. Thus, while the prediction of amino acid exchanges on substrate metabolism remain difficult, folding stability of variant proteins of interest can be estimated using existing computational tools based on sequence homology modeling (Kulshreshtha et al., 2016).

## Evaluation of Truncation Variants

Drug metabolizing enzymes and transporters have been found to harbor a multitude of truncation variants, such as micro-insertions and micro-deletions (indels) causing frameshifts, stop-gain and start-lost variants. Some of these variants are clinically relevant and occur with high frequencies in specific populations, including the stop-gain variant CYP2C19^*^3 in East Asians and the frameshift variants CYP2D6^*^3 and CYP2D6^*^6 in Europeans (Zhou et al., 2017). As most pharmacogenes have only minor endogenous functions, they are under low evolutionary pressure and, consequently, such loss-of-function variants are often not selected against (Lauschke et al., 2017). Moreover, it has been speculated that pharmacogenetic loss-of-function alleles can even be selected for in modern humans, possibly due to reduced bioactivation of dietary toxicants (Fujikura, 2016). Truncation variants are commonly assumed to have deleterious effects and only few studies have been presented that provide approaches to quantitatively assess the functional consequences of such mutations (Cline and Karchin, 2011).

Early bioinformatic tools, such as LOFTEE, prioritize truncation variants based on a set of empirical rules, including whether the variant of interest occurs in the last 5% of transcript or whether the truncating allele is the ancestral state (MacArthur et al., 2012). Other approaches, such as Likelihood-ratio scoring (Zia and Moses, 2011), SIFT Indel (Hu and Ng, 2012) and NutVar (Rausell et al., 2014), primarily utilize the evolutionary conservation of amino acid residues. However, predictive performance of these tools for loss-of-function mutations is limited when trained on only missense mutations. Moreover, these methods are trained on genes that have high-quality annotations, which poses problems for the functional interpretation of truncation variants in genes for which such annotations are not readily available.

To overcome these shortcomings, CADD was developed by integrating many diverse functional genomics annotations into a single score for each variant, which allows to estimate the impact of all classes of genetic variation, including truncating variants (Kircher et al., 2014). Newer approaches, such as DDIG-in (Folkman et al., 2015) and VEST-Indel (Douville et al., 2016) supplement conservation-based features with information about sequence and structural properties at nucleotide and protein levels as well as intrinsic disorder predictions from the region affected by stop gain and frameshift variants. Notably, the recently developed tool ALoFT (Annotation of Loss-of-Function Transcripts) can categorize the pathogenic importance of putative loss-of-function mutations by integrating variant information with redundancy and haplosufficiency data of the corresponding gene (Balasubramanian et al., 2017). However, aforementioned methods are primarily focused on distinguishing benign and disease-causing mutations. Thus, future studies are needed to evaluate whether this emphasis on the pathogenicity of variants might affect the performance of these methods regarding the functionality prediction of truncating variants in genes not associated with disease, such as many ADME genes.

In addition to impacts on functional and structural properties of proteins, truncating variants can affect nonsense-mediated mRNA decay (NMD). NMD is a conserved translation-dependent mechanism that is responsible for recognizing and eliminating aberrant mRNA transcripts to prevent the production of truncated peptides, thereby playing a critical role in preventing the accumulation of misfolded protein and subsequent initiation of the unfolded protein response (UPR) (Kervestin and Jacobson, 2012; Schoenberg and Maquat, 2012). Recently, Hsu et al. presented NMD Classifier, a tool for the systematic classification of NMD events, which was reported to correctly identify 99.3% of the NMD-causing transcript structural changes (Hsu et al., 2017). The incorporation of this information alongside functional estimates is expected to not only increase discriminative power but also to suggest the nature of the functional impact of a given variant. Interestingly, there is evidence that NMD efficiency varies between individuals and that these differences correlate with response to NMD inhibitors in cystic fibrosis patients (Linde et al., 2007; Kerem et al., 2008). While this phenomenon has to the best of our knowledge not been explicitly tested in the context of pharmacogenomics, inter-individual differences in NMD magnitude could, at least in part, explain the large differences in drug response between patients with loss-of-function genotypes (Jukić et al., 2018) and thus have important implications for therapy.

In summary, much progress has been made regarding the functional interpretation of variants causing truncations of the corresponding gene product and current computational tools are able to incorporate a variety of features into their predictions, including evolutionary conservation, sequence and structural information as well as putative effects on NMD. However, it remains to be demonstrated whether these available tools will also be suitable for the prediction of effects of truncation variants in poorly conserved pharmacogenetic loci.

## Prediction of Aberrant Splicing Events

Splicing of pre-mRNA is a critical step during mRNA maturation in which introns are excised and exons are ligated. This process necessitates the presence of 5′ and 3′ splicing signals and branch point sequence and is further regulated by exonic and intronic splicing enhancer/silencer (ESE/ESS and ISE/ISS, respectively) (Lee and Rio, 2015; Shi, 2017). Mutations in these regions can disrupt the splicing process and result in aberrantly processed transcripts, which can trigger NMD or result in the production of dysfunctional proteins. The functional importance of genetic variants in splice sites is emphasized by estimates that around 15% of human pathogenic mutations cause dysregulation of splicing (Baralle et al., 2009).

Variants located in canonical splice sites are considered having the largest effect on splicing events. Therefore, a multitude of computational algorithms were developed to handle the prediction of 5′ and 3′ splice site, such as NNSplice (Reese et al., 1997), MaxEntScan (Yeo and Burge, 2004), GeneSplicer (Pertea et al., 2001), and SplicePort (Dogan et al., 2007; Table 3). Moreover, variants outside splice sites can have substantial effects on splicing (Soukarieh et al., 2016) and a variety of computational methods have been developed to predict the effect of such regulatory sequences. Examples are sequence the conservation-based algorithm Skippy (Woolfe et al., 2010) and the machine learning tools MutPred Splice (Mort et al., 2014), scSNVEL (Jian et al., 2014b), SPANR (Xiong et al., 2015), and CryptSplice (Lee et al., 2017). Further tools are available for the identification of branch point sequences (Corvelo et al., 2010; Zhang et al., 2017). Lastly, the secondary structure of pre-mRNAs can interfere with splice-site recognition, modulate spliceosome binding or can facilitate splicing efficiency by bringing splice donors and acceptors into close proximity (Warf and Berglund, 2010). Consequently, genetic variants that alter pre-mRNA structure were found to promote alternative splicing (Wan et al., 2014), incentivizing the incorporation of structural information provided by tools, such as TurboFold (Harmanci et al., 2011) or CentroidFold (Sato et al., 2009), into variant effect predictions. For a more detailed description of structural RNA analyses we refer the interested reader to excellent recent reviews (Jian et al., 2014a; Lorenz et al., 2016; Ohno et al., 2018).

**Table 3:** Tools for the prediction of variant effects on splicing, transcript levels or translation.

**Algorithm**	**Application**	**Basis of decision**	**Model training or evaluation**	**References**
NMD Classifier	NMD	Prediction of NMD for a given transcript based on comparison to most similar coding transcript	Simulation-based evaluation based on screening artificial transcript structure-altering events	Hsu et al., 2017
NNSplice	Splicing (splice sites)	Sequence splice site analysis using HMM	Distinguish splice site sequences from sequences in the neighborhood of real splice sites	Reese et al., 1997
MaxEntScan	Splicing (splice sites)	Splice site analysis by modeling short sequence motifs using the maximum entropy principle with constraints estimated from available data.	1,821 transcripts unambiguously aligned across the entire coding region, spanning a total of 12,715 introns	Yeo and Burge, 2004
GeneSplicer	Splicing (splice sites)	Splice site prediction using maximal dependence decomposition with the addition of markov model to capture dependencies among neighboring bases	Annotated genes from the Exon-Intron Database	Pertea et al., 2001
SplicePort	Splicing (splice sites)	Splice site prediction using C-modified least squares learning based on positional and compositional sequence features	Training on 4,000 pre-mRNA human RefSeq sequences and test on B2Hum data set	Dogan et al., 2007
Skippy	Splicing (regulatory sequences)	Prediction of variants causing exon skipping, exon inclusion or ectopic splice site activation based on sequence information, proximity to splice junctions and evolutionary constraint of the peri-variant region	Multiple exonic splicing regulatory elements datasets as positive data and HapMap variants as splicing-neutral variants	Woolfe et al., 2010
MutPred Splice	Splicing (regulatory sequences)	Prediction of auxiliary splice sequences using multiple variant-, flanking exon- and gene-based features	Splicing variants from HGMD as pathogenic set and non-splicing variants from both HGMD and 1000G as neutral controls	Mort et al., 2014
scSNVEL	Splicing (splice sites)	Ensemble prediction using 8 algorithms using random forest learning	Splice variants from HGMD, SpliceDisease database and DBASS as pathogenic set and variants not implicated in splicing from both HGMD and 1000G as controls	Jian et al., 2014b
SPANR	Splicing (splice sites and splice regulatory sequences)	Integrating 1,393 sequence features from each exon and its neighboring introns and exons to identify splice sites as well as intronic and exonic splice regulators	10,689 exons that displayed evidence of alternative splicing	Xiong et al., 2015
CryptSplice	Splicing (splice sites)	Prediction of cryptic splice-site activation using an SVM model	Sequences from the annotated NN269 and HS3D splice datasets with positive sequence in splice sites and control sequence outside splice sites	Lee et al., 2017
Corvelo *et al*.	Splicing (branch points)	Analysis of splice site sequence conservation and position bias using SVM	A set of 8,156 conserved putative branch point sequences from 7 mammalian species	Corvelo et al., 2010
BPP	Splicing (branch points)	Identification of branch point motifs by integrating information on the branch point sequence and the polypyrimidine tract	Intron sequences longer than 300 nucleotides	Zhang et al., 2017
TurboFold	Splicing (pre-mRNA structure)	Probabilistic method that integrates comparative sequence analyses with thermodynamic folding models	Thorough benchmarking against three methods that estimate base pairing probabilities and eight tools for structural predictions based on known RNA structures	Harmanci et al., 2011
CentroidFold	Splicing (pre-mRNA structure)	RNA secondary structure prediction using the γ-centroid estimator	Validation based on 151 RNA experimentally determined RNA structures	Sato et al., 2009
mrSNP	miRNA binding	miRNA binding energy calculations for reference and variant containing sequence and report of binding difference	Evaluation based on variants that map to miRNA targets predicted by TargetScan	Deveci et al., 2014
PinPor	RBP binding	Bayesian network approach that incorporates information about sequence features, stabilization of RNA secondary structure and evolutionary conservation	Inframe indels from HGMD as pathogenic and common indels from 1000G as neutral controls	Zhang et al., 2014

*HGMD, Human Gene Mutation Database; 1000G = 1000 Genomes Project; DBASS, Database for Aberrant Splice Sites; NMD, nonsense-mediated decay; HMM, hidden Markov model; RBP, RNA binding protein*.

In ADME genes, dysregulation of splicing has long been recognized as a cause for inter-individual variability drug metabolism (Hanioka et al., 1990) and toxicity (Raida et al., 2001) and the liver was found to be is among the tissues with highest levels of alternative splicing activity (Yeo et al., 2004). As splicing is highly tissue specific, these data indicate that algorithms for the prediction of variant splice effects in pharmacogenetics should ideally be trained on positive control sets for which aberrant splicing is confirmed in the tissue of interest, i.e., primarily liver. To this end, the GTEx project (GTEx Consortium, 2017) provides a rich resource that has already been successfully utilized for the identification of tissue-specific splice events in pharmacogenes (Chhibber et al., 2017).

In summary, the toolkit of available computational algorithms for the prediction of variant effects on splicing has rapidly grown and by now allows not only to evaluate direct impact on splice sites, but also to assess mutations in regulatory splice enhancers and silencers, as well as branch points. For the application of these methods for pharmacogenomics there is a need to benchmark available tools on splice variants in ADME genes. Moreover, we anticipate that the utilization of tissue-specific expression data will further refine splice site predictions.

## Functional Impact of Variants in Untranslated Regions

miRNAs play important roles in the regulation of mRNA stability and translation. miRNA-mRNA interaction occurs through conserved miRNA binding sites in the 3′-UTRs and at least 10% of all SNPs are located in 3′-UTRs and might affect complementary miRNA-mRNA pairing (Xiao et al., 2009). Furthermore, miRNAs have been shown to be important modulators of ADME gene expression profiles (Rieger et al., 2013). Therefore, functional interpretation of genetic variations within miRNA target sites constitutes an important factor for the prediction of the fate of corresponding transcript. Thus, to evaluate the potential relevance of genetic polymorphisms in UTRs various databases, such as the polymiRTS Database 3.0 (Bhattacharya et al., 2014) or MirSNP (Liu et al., 2012), provide useful resources that contains a collection of experimentally confirmed SNPs and indels not only in miRNA target sites but also in miRNA seed regions responsible for mRNA binding. Furthermore, a variety of other SNP effect prediction servers are publically available (Fehlmann et al., 2017).

In case no experimental data is available, various computational tools can be used to predict possible disruption of the miRNA-mRNA pairing for a given variant (Table 3). MicroSNiPer (Barenboim et al., 2010) and ImiRP (Ryan et al., 2016) identify and predict such disruptions by comparing the mutant 3′-UTR sequences with major variant databases. Similarly, mrSNP can predict the effect of any variant identified in NGS-based projects on miRNA-target transcript interaction (Deveci et al., 2014). However, it is important to note that miRNA target predictions seem to have a high false-positive rate (Pinzón et al., 2017), suggesting that these problems might be lingering for studies utilizing miRNA-target databases without stringent experimental validations. Besides predicting the effect of genetic variants in putative miRNA target sites, multiple online tools are available for inverse approaches, analyzing variants in miRNAs or pre-miRNAs for possible deleterious effects. For more comprehensive collection of miRNA related variant interpretation tools the reader is referred to the recent reviews and online resources (Akhtar et al., 2016; Moszynska et al., 2017).

In addition, recent approaches expanded the methodological portfolio beyond miRNA binding site prediction to include effects of UTR variants on binding of RNA-binding proteins (RBPs), translational efficacy and ribosomal loading. Effects of indels on RBP binding can be evaluated using PinPor, which has been demonstrated to have some success in distinguishing disease-causing and neutral indels (Zhang et al., 2014). Furthermore, Sample *et al*. presented the preprint of a deep learning approach based on experimental polysome profiling to predict the impact of UTR sequence on translation (Sample et al., 2018). These developments nicely indicate the diversification of parameters that can incorporated into variant effect predictions, thus further refining biological interpretation of NGS data sets.

## Analysis of Regulatory Variants

Non-coding regions account for more than 99% of the human genome and, consequently, their consideration substantially expands the analysis space of computational predictions. Variants in non-coding regions can affect regulatory elements, such as promoters, enhancers, silencers, and insulators, which, in turn, may alter their affinity to transcription factor or remodel the local chromatin structure (Zhang and Lupski, 2015; Deplancke et al., 2016). Accurate prediction of the functional consequences of such variants constitutes one of the major challenges in human genetics.

To interpret noncoding variants, a variety of different strategies have been presented. The first approaches, such as SiPhy (Garber et al., 2009), PhyloP (Pollard et al., 2010), PhastCons (Siepel et al., 2005), GERP++ (Davydov et al., 2010), or SCONE (Asthana et al., 2007), were based on evolutionary constraint using sequence alignments. However, the observation that no enhanced constraints were identified in regulatory elements at the level of DNA sequence despite conserved transcription factor binding led to the realization that conservation of regulatory regions can only be a weak indicator of the functional effects of SNVs in regulatory regions (Schmidt et al., 2010; Arbiza et al., 2013). Consequently, conservation metrics were complemented with additional functional genomics features, such as the sequence and genic context, transcription factor binding profiles (Johnson et al., 2007), histone modification data (Zhang et al., 2010) and DNase I hypersensitive sites (Boyle et al., 2008) in an attempt to improve prediction quality. Based on these rich data sets, a variety of ensemble classifiers were developed using various machine learning approaches that aim to distinguish neutral from pathogenic variants, including GWAVA (Ritchie et al., 2014), CADD (Kircher et al., 2014), FATHMM (Shihab et al., 2013, 2015; Rogers et al., 2018), DANN (Quang et al., 2015), DIVAN (Chen et al., 2016), and Genomiser (Smedley et al., 2016) (Table 4).

**Table 4:** Algorithms for the functional interpretation of regulatory variants.

**Algorithm**	**Model**	**Application**	**Model training**	**Features**	**References**
FATHMM	HMM	Pathogenic variants	HGMD regulatory variants as pathogenic set and common 1000G variants as controls	Evolutionary conservation data (PhastCons and PhyloP), chromatin accessibility (DNase-HSS and FAIRE-Seq), TF binding and histone modification ChIP-Seq data, genome segmentation, frequency data (1000G and ESP) and information about genic and sequence context	Shihab et al., 2013, 2015; Rogers et al., 2018
GWAVA	RF	Pathogenic variants	HGMD regulatory variants as pathogenic set and common 1000G variants as controls	Evolutionary conservation data (GERP), chromatin accessibility (DNase-HSS and FAIRE-Seq), TF binding and histone modification ChIP-Seq data, genome segmentation, frequency data (1000G) and information about genic and sequence context	Ritchie et al., 2014
CADD	SVM	Deleterious variants	Sites with MAF < 5% where for which the human genome differed from the inferred human-chimp ancestral genome and equal number of simulated variants	Evolutionary conservation data (GERP++, PhastCons and PhyloP), chromatin accessibility (DNase-HSS and FAIRE-Seq), TF binding and histone modification ChIP-Seq data, genome segmentation, frequency data (1000G and ESP) and information about genic and sequence context	Kircher et al., 2014
DANN	NN	Deleterious variants	Same as CADD but using deep neural networks instead of linear SVM.		Quang et al., 2015
DeepSEA	NN	Variants that affect gene expression	HGMD regulatory variants, eQTLs and NHGRI GWAS phenotype-associated SNPs	Evolutionary conservation data (GERP++, PhastCons and PhyloP), chromatin accessibility (DNase-HSS and FAIRE-Seq), TF binding and histone modification ChIP-Seq data	Zhou and Troyanskaya, 2015
gkm-SVM	SVM	Variants that affect gene expression	Tissue-specific enhancer sequences marked by H3K4me1 from length-, GC content- and repeat-matched random control	Definition of tissue-specific regulatory dictionary based on chromatin accessibility (DNase-HSS) and H3K4me1 ChIP-Seq data	Lee et al., 2015
fitCons	INSIGHT	Prediction of *cis*-regulatory elements	Unsupervised classifier that clusters genomic regions on the basis of functional genomic data and then estimates a probability of fitness consequences for each group from associated patterns of genetic polymorphism and divergence.	Evolutionary conservation data (GERP, PhastCons and PhyloP), chromatin accessibility (DNase-HSS), TF binding and histone modification ChIP-Seq data, genome segmentation and RNA-Seq data	Gulko et al., 2015
GenoCanyon	US	Identification of functional regions	Unsupervised classifier based on the estimated proportion of functional regions in the human genome.	Evolutionary conservation data (GERP and PhyloP), chromatin accessibility (DNase-HSS and FAIRE-Seq), TF binding and histone modification ChIP-Seq data	Lu et al., 2015
DIVAN	EL	Disease-specific risk variants	Disease-specific regulatory NHGRI GWAS SNPs and common 1000G variants or benign GWAS SNPs as controls	Chromatin accessibility (DNase-HSS and FAIRE-Seq), TF binding and histone modification ChIP-Seq data	Chen et al., 2016
Genomiser	RF	Mendelian disease	Sites with MAF < 5% where for which the human genome differed from the inferred human-chimp ancestral genome as functionally neutral variation and 453 positive variants based on literature review	Evolutionary conservation data (GERP++, PhastCons and PhyloP), chromatin accessibility (DNase-HSS), TF binding and histone modification ChIP-Seq data, frequency data (1000G and ESP) and information about enhancer context from FANTOM5	Smedley et al., 2016
Eigen	US	Effect of variants on gene expression and disease risk	Unsupervised classifier based on the blockwise conditional independence between annotations given the functional impact of the variant.	Evolutionary conservation data (GERP, PhastCons and PhyloP), chromatin accessibility (DNase-HSS and FAIRE-Seq), TF binding and histone modification ChIP-Seq data and frequency data (1000G)	Ionita-Laza et al., 2016

*RF, random forest; SVM, support vector machine; HMM, hidden Markov model; EL, ensemble learning; NN, neural networks; INSIGHT, Inference of Natural Selection from Interspersed Genomically Coherent Elements Gronau et al., 2011; US, unsupervised; HGMD, Human Gene Mutation Database; 1000G, 1000 Genomes Project; ESP, Exome Sequencing Project; TF, transcription factor; HSS, hypersensitive site; FAIRE, Formaldehyde-Assisted Isolation of Regulatory Elements Giresi et al., 2007; NHGRI, National Human Genome Research Institute*.

In contrast, other methods, such as gkm-SVM (Lee et al., 2015) and DeepSEA (Zhou and Troyanskaya, 2015) have been developed to predict regulatory elements based on primary sequence alone. Trained on publically available cell type-specific chromatin data provided by ENCODE (The ENCODE Project Consortium, 2012) and the Roadmap Epigenomics Project (Roadmap Epigenomics Consortium et al., 2015) as well as transcription factor binding patterns accessible via JASPAR (Khan et al., 2018), these algorithms predict to what extent a genetic variant will cause changes to the local chromatin profiles and how these effects translate into functional consequences. The resulting data demonstrate that inferring consequences from functional genomics data is highly cell type and context specific and relies on biologically appropriate training sets. These convincing findings incentivize the generation of functional genomics data from carefully phenotyped human tissues involved in drug ADME to derive tissue-specific regulatory lexica and we envision that training machine learning approaches on these data sets will substantially increase the power of regulatory pharmacogenetic prediction classifiers.

As with coding variants, the use of potentially biased training sets and multi-dimensional circularity between training and test data constitutes an inherent problem for current variant prediction tools (Grimm et al., 2015). For instance, a variety of algorithms consider common variants from the 1000 Genomes project as functionally neutral control sets for model training. However, while these variants are likely to be depleted of pathogenic variants in haploinsufficient genes, many common variants entail functional consequences in their respective gene product, particularly if the gene is rapidly evolving, such as many *CYP* genes. Similar problems arise when the model is trained using phenotype associated GWAS polymorphisms as functional variant sets, as only 5.5% of GWAS index SNPs are estimated to be causal whereas the remainder is only in linkage disequilibrium with the true functional variant in the locus (Farh et al., 2015).

To overcome these problems, unsupervised approaches have been developed that do not rely on the labeling of training data, thereby reducing the dependence on preexisting variant classifications and existing models of mutation. These unsupervised models, such as GenoCanyon (Lu et al., 2015) and Eigen (Ionita-Laza et al., 2016), represent powerful tools for the genome-wide interpretation of variants. However, as they are calibrated on genome-wide data, it remains to be determined whether gene class-specific peculiarities, such as low evolutionary conservation in ADME genes, might affect the predictive accuracy of these approaches for pharmacogenetic applications.

## Conclusions

Technical progress in NGS technology has resulted in its routine application in medical genetics and clinical diagnostics. In contrast, clinical implementation of NGS-based pharmacogenomics is largely lagging behind (Lauschke and Ingelman-Sundberg, 2016b; Ji et al., 2018). Most importantly, in order to utilize the major advantage of NGS-based genotyping, which is the discovery of the entire panorama of the individual's genetic portfolio, tools have to be in place, which allow to translate these variability data into functional consequences and clinical recommendations. Whereas, the identification of rare putatively deleterious mutations in congenital diseases is aided by clear phenotypic alterations of the affected patient and the possibility to perform comparative genomic analyses of unaffected family members, pharmacogenomic phenotypes are generally more difficult to detect as they only present in a given context, such as exposure to specific medications. In the absence of drug response associations or experimental characterizations that support the functional interpretation of rare variants, there is thus an urgent need for reliable computational prediction tools to fill this space.

Importantly, recent developments in computational variant effect prediction methods promise to narrow the gap to meet the exacting demands on genomics applications in the clinics. Machine learning constitutes an important tool kit to fully harness the power of large data sets provided by NGS. However, these approaches rely on accurate labeling of input variants, i.e., training data need to be correctly classified into deleterious and functionally neutral variants. Thus, we advocate for approaches that leverage smaller data sets of variants for which comprehensive experimental or functional genomic data is available instead of training algorithms on large but functionally poorly annotated data, such as treating all common polymorphisms identified in the 1000 Genomes Project as functionally neutral. In addition, we endorse previous appeals for the sharing of codes and data sets, which will enable comparative benchmarking of newly developed tools and algorithms and will accelerate research progress within the area of computational pharmacogenomics and beyond (Kalinin et al., 2018).

The functional consequences of missense variants have been most extensively studied. Respective methods base their predictions on evolutionary conservation and structural information of the polypeptide encoded by the respective gene. Importantly, while evolutionary conservation is a suitable measure to inform about the deleteriousness of a variant, i.e., its effect on organismal fitness, it is not suitable for the prediction of variant effects in genes under low selective pressure, such as most pharmacogenes. Recognition of these conceptual problems resulted in the development of computational predictors trained specifically on ADME missense variants (Zhou et al., 2018). We envision that these approaches will become more powerful with increasing functionally annotated pharmacogenetic variant data.

Furthermore, multiple strategies have been developed to analyze the functional impact of variants in non-coding regions of the genome, which are increasingly recognized as a substantial contributor to inter-individual variability. An increasing number of algorithms is by now available that base their predictions on a multitude of different parameters, including effects on miRNA binding or translational efficiency, modulation of splicing and impacts on transcriptional events by disruption of transcription factor binding sites or polymerase loading (Figure 1). While these developments provide a methodological arsenal to comprehensively characterize all different classes of genetic variants, these methods are generally trained on pathogenic variant sets and have not been benchmarked on independent data sets. Thus, their predictive power for pharmacogenetic assessments remains to be evaluated.

**Figure 2:**
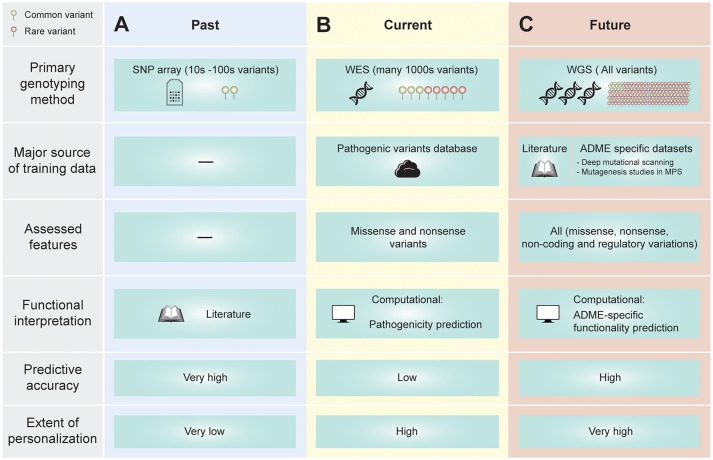
The past, present and future of pharmacogenetic phenotype predictions. **(A)** Conventionally, pharmacogenetic predictions were based on the interrogation of few common candidate SNPs, whose functional effects were predicted based on extensive literature evidence, resulting in high predictive accuracy but only few considered variations. **(B)** With increasing prevalence of whole exome sequencing (WES), a multitude of pharmacogenetic variants with unknown functional relevance are identified. These variants can be interpreted using computational methods. However, current algorithms are generally trained to detect the pathogenicity rather than the functionality of queried variants, resulting in overall relatively low predictive accuracy. Furthermore, only effects of missense and nonsense variants are evaluated. **(C)** In the near future, whole genome sequencing (WGS) will become the predominant genotyping methodology, revealing not only coding variants but also variants in regulatory regions and introns. To facilitate interpretation of this data, we envision that pharmacogenetic predictors will be directly trained on functionally annotated ADME data sets. Emerging technologies, such as deep mutational scanning for the systematic interrogation of missense variants or mutagenesis screens in microphysiological systems (MPS) for the characterization of variants in regulatory regions, provide powerful tools to generate these data, boosting the predictive performance of data hungry machine learning tools. These advances allow to go beyond the interpretation of missense and nonsense variants and to include also non-coding and regulatory variations into pharmacogenetic assessments.

The prediction of drug metabolism phenotypes based on the genotype of the individual has made tremendous progress over the last decades (Figure 2). Conventional approaches use data from few candidate variants for which substantial *in vitro* or *in vivo* characterization data was available to predict drug response. While this strategy has been successful in incorporating common pharmacogenetic variability into clinical decision-making, they fail to address functional effects of the vast extent of rare genetic variants. To also include rare variants, pilot programs were initiated in which WES was used to comprehensively interrogate the genetic landscape of pharmacogenomic loci (Bielinski et al., 2014). However, analyses were restricted to pharmacogenetic missense variants and the effects of SNVs with unknown functional relevance were interpreted using computational models trained on pathogenic data sets with negative impacts on the accuracy of phenotype predictions, as discussed above. Thus, while these strategies constitute an important step toward the further personalization of genotype-guided treatment decisions their predictive accuracy is rather low.

We expect that technological, methodological and analytical progress will contribute to a further refinement of NGS-guided drug treatment in the near future. Firstly, technological advances will result in an increasing dissemination of WGS, which facilitates the incorporation of the entire profile of an individual's genetic variability, including regulatory variants, into pharmacogenetic predictions. Secondly, we envision that novel high-throughput methodologies for functional characterizations, such as deep mutational scanning, will provide powerful approaches to generate large functionally annotated pharmacogenetic variant data sets. In addition, recent advances in the development of microphysiological systems (MPS) that allow to model key target tissues associated with drug metabolism or safety provide (Ewart et al., 2018) provide promising tools to generate tissue-specific and human-relevant data sets for studies of gene-drug interactions (Ingelman-Sundberg and Lauschke, 2018). Using this integrated wealth of functional pharmacogenetic data to train machine learning models aspires to provide high-accuracy predictions based on the entire genetic variability landscape of the respective patient.

Importantly, leveraging this information as guidance for clinical decision-making promises to increase treatment efficacy and reduce the risks of adverse events in carriers of pharmacogenetic variants whose effects have not been experimentally evaluated. Current market analysis estimates suggests that implementation of artificial intelligence into the clinical decision support toolbox might increase average life expectancy in the Western World by 0.2–1.3 years and reduce total health care expenditures by 5–9%, corresponding to 2 trillion to 10 trillion USD globally per year (Bughin et al., 2017). However, in order to realize these exciting prospects, there is a need for prospective, randomized controlled trials that evaluate patient outcomes and cost-effectiveness of such preemptive advice across genes, drugs and health care systems.

In summary, computational prediction methods are essential for the implementation of NGS into clinical decision-making. While much progress has been made and a plethora of conceptually diverse tools is already available, there is a need to develop specialized methods that are optimized for the prediction of variant functionality rather than pathogenicity and are calibrated specifically on pharmacogenetic data. We envision that technological, methodological and analytical advances will soon allow to comprehensively predict variant effects with sufficient accuracy to justify the design of trials in which the clinical value of NGS-guided treatment decisions can be tested in a prospective setting.

## Author Contributions

All authors listed have made a substantial, direct and intellectual contribution to the work, and approved it for publication.

### Conflict of Interest Statement

VL is co-founder and owner of HepaPredict AB. The remaining authors declare that the research was conducted in the absence of any commercial or financial relationships that could be construed as a potential conflict of interest.
